# Cellular immune response to SARS-CoV-2 in patients with primary antibody deficiencies

**DOI:** 10.3389/fimmu.2023.1275892

**Published:** 2023-10-12

**Authors:** Dorota Mizera, Radosław Dziedzic, Anna Drynda, Ada Gradzikiewicz, Bogdan Jakieła, Magdalena Celińska-Löwenhoff, Agnieszka Padjas, Aleksandra Matyja-Bednarczyk, Lech Zaręba, Stanisława Bazan-Socha

**Affiliations:** ^1^ Center for Innovative Medical Education, Jagiellonian University Medical College, Kraków, Poland; ^2^ Doctoral School of Medical and Health Sciences, Jagiellonian University Medical College, Kraków, Poland; ^3^ Students’ Scientific Group of Immune Diseases and Hypercoagulation, Jagiellonian University Medical College, Kraków, Poland; ^4^ Department of Internal Medicine, Faculty of Medicine, Jagiellonian University Medical College, Kraków, Poland; ^5^ College of Natural Sciences, Institute of Computer Science, University of Rzeszow, Rzeszów, Poland

**Keywords:** primary antibody deficiencies, PAD, COVID-19, interferon-gamma release assay, cellular immune response

## Abstract

**Introduction:**

Primary antibody deficiencies (PAD) are inborn defects of the immune system that result in increased susceptibility to infections. Despite the reduced response to vaccination, PAD patients still benefit from it by reducing the risk of severe infections and complications. SARS-CoV-2 vaccines are recommended in PAD patients, but their immune effects are poorly studied. Here, we analyze virus-specific T-cell responses in PAD patients after booster vaccination against SARS-CoV-2.

**Patients and methods:**

The study included 57 adult PAD patients on long-term immunoglobulin replacement therapy (IgRT) diagnosed with X-linked agammaglobulinemia (XLA; n = 4), common variable immunodeficiency (CVID; n = 33), isotype defects or IgG subclass deficiency (n = 6), and unclassified IgG deficiency (n = 14). Of those, 49 patients (86%) received vaccination against SARS-CoV-2 using mRNA vaccine (Pfizer-BioNTech). T-cell responses were assessed at a median of 21 (13 – 30) weeks after the booster dose (mainly the third dose) using commercially available interferon-gamma release assay (IGRA) with recombinant SARS-CoV-2 spike S1 protein.

**Results:**

Vaccinated PAD patients showed an increased (3.8-fold, p = 0.004) release of IFN-γ upon S1 stimulation. In this group, we also documented higher serum levels of anti-SARS-CoV-2 IgG (4.1-fold, p = 0.01), although they were not associated with IGRA results. Further subgroup analysis revealed very similar IGRA responses in CVID and unclassified IgG deficiencies that were 2.4-fold increased compared to XLA and 5.4-fold increased compared to patients with isotype defects or IgG subclass deficiencies (e.g., vs. CVID: p = 0.016). As expected, CVID and XLA patients showed decreased serum titers of anti-SARS-CoV-2 antibodies compared to other studied groups (e.g., CVID vs. unclassified IgG deficiency: 4.4-fold, p = 0.006). The results did not depend directly on IgRT mode or dose, number of vaccine doses and time from the last booster dose, and clinical manifestations of PAD. Interestingly, anti-SARS-CoV-2 titers were positively correlated with serum immunoglobulin levels before IgRT (e.g., for IgA: r = 0.45, p<0.001; for IgG: r = 0.34, p = 0.009) and the percentage of peripheral blood NK cells (r = 0.48, p<0.001).

**Conclusions:**

Our results documented satisfactory *in vitro* cellular immune response in PAD patients after booster SARS-CoV-2 vaccination. Therefore, even patients with agammaglobulinemia should benefit from vaccination due to the apparent induction of cell-mediated immunity, which, together with IgRT, grants comprehensive protection against the pathogen.

## 1 Introduction

Primary immunodeficiencies (PID) are a heterogeneous group of inborn disorders associated with various functional defects of the immune system. As different components of immunity can be affected, patients are generally prone to severe and recurrent infections. They experience treatment burdens, chronic complications, and reduced life expectancy (1, 2). Primary antibody deficiencies (PAD) represent the most common group of inborn errors of immunity, accounting for more than 50% of PID cases worldwide (3). Patients with PAD are characterized by an inability to mount effective humoral responses due to variable defects in B-cell function and/or antibody production, resulting in a variety of manifestations, from a marked decrease in circulating B-cell number and serum immunoglobulins to specific antibody deficiencies (4). That entails a spectrum of clinical symptoms, with characteristic recurrent respiratory tract infections, but also leads to various non-infectious complications resulting from aberrant lymphoproliferation and autoimmunity (5). PAD therapy depends on a specific type of disorder, but is primarily focused on preventing recurrent infections, including long-term antimicrobial prophylaxis and immunoglobulin replacement therapy (IgRT) (6, 7).

Respiratory viral infections are common in patients with PAD (8). They are associated with more severe symptoms and longer viral clearance, often challenging even in patients with adequately administered IgRT (9). Likewise, SARS-CoV-2 infections in PAD were characterized by a more severe course, with approximately 50% requiring hospitalization and an overall fatality rate of 9% (10, 11). Furthermore, it has been shown that PAD patients may present with prolonged infections, measured by the time from the first positive SARS-CoV-2 test to the first negative test (11). However, the burden of COVID-19 symptoms depended largely on the stage of the pandemic and the virulence of the predominant SARS-CoV-2 strains (12). As of 2023, the number of severe cases in the general population constantly decreases due to vaccination programs and novel virus variants causing milder disease manifestations. Nevertheless, COVID-19 still represents a significant public health problem, including unfavorable sequels and the ‘long-COVID’ cases observed even after milder disease (13).

The increased risk of severe disease and death from COVID-19 in patients with PID raised the need to introduce safe and effective vaccination measures targeting this cohort (14, 15). Earlier studies indicated that PAD patients showed at least some immune response to COVID-19 vaccination; however, its effectiveness in reducing hospitalization rate was lower than in the general population (11). For example, Pham et al. (16) demonstrated that two doses of the mRNA vaccine resulted in a positive cellular response in 77% of patients with PAD. Yet, specific and functional IgG antibodies were detected only in a minority of these subjects. It is now accepted that vaccination in patients with PAD is safe (17). In many cases, it leads to an improvement in the immune response against the virus and a reduction in severe complications related to infection (18). However, its effectiveness in different PAD subtypes has not been studied in detail.

We hypothesized that the effectiveness of the COVID-19 vaccine in the adult PAD population depends on the disease subtypes, differing in the extent to which cellular and humoral immune responses are compromised. To validate this hypothesis, we evaluated responses to vaccination against the SARS-CoV-2 in PAD patients with different disease subtypes and clinical manifestations using a validated interferon-gamma release assay (IGRA) that measures the release of IFN-γ by peripheral blood mononuclear cells stimulated *in vitro* with the SARS-CoV-2 S (spike) 1 protein (19, 20).

## 2 Methods

### 2.1 Patients and study design

The study included 57 patients with a confirmed diagnosis of PAD who underwent chronic IgRT for at least one year at the Outpatient Clinic of the Immunology Department (University Hospital, Krakow, Poland). The diagnosis of PID was based on the criteria of the European Society for Immunodeficiencies (ESID) (21). The leading secondary causes of immunodeficiency, including neoplastic or hematologic disorders and human immunodeficiency virus infection, were excluded. Demographic and clinical characteristics were recorded at enrollment based on a structured questionnaire and health records. Certain data, such as the lowest IgG, IgA, and IgM levels (i.e., at the diagnosis), were based on medical history. Similarly, major lymphocyte subpopulations (e.g., T-cell subsets, B-cells, and NK cells) were routinely quantified using flow cytometry (FACS Canto II; Franklin Lakes, USA) at enrolment or in the majority during the last 12 months. Serum immunoglobulin concentrations were determined by nephelometry (BN II System; Siemens Healthineers, Erlangen, Germany). IgG trough levels were expressed as the mean of the three separate measurements every four consecutive weeks prior to study enrolment.

The patients were recruited between January and August 2022, corresponding to the third wave of COVID-19 in Poland, with infections caused predominantly by Omicron 21K and 21L variants of SARS-CoV-2 (22). We enrolled 57 consecutive PAD patients on long-term IgRT. Exclusion criteria were as follows: pregnancy, breastfeeding, confirmed COVID-19 or any other ongoing infection, active neoplastic disease, congestive heart failure (WHO class III/IV), liver injury (i.e., increase in serum alanine transaminase activity >2-fold the normal upper range), kidney insufficiency (estimated glomerular filtration rate < 60 mL/min/1.73 m^2^), hyper- or hypothyroidism, diabetes mellitus, and current use of systemic steroids or immunosuppressants. The number of confirmed SARS-CoV-2 infections in the past and the status of vaccination was assessed retrospectively based on the patient’s history and the registry of the Polish National Vaccination Program. Of all PAD patients enrolled, 49 (86%) received mRNA vaccine (Pfizer-BioNTech) with two (35%), three (59%), or four (6%) doses in total. Only eight patients (all with CVID) refused vaccination. COVID-19 in medical history was reported in 18 (32%) patients with a median of 29 (21 – 72) weeks before the study enrollment, with no difference between vaccinated and non-vaccinated groups. Only in 5 cases the disease occurred after the last vaccination dose, yet with relatively mild clinical symptoms. The study started after SARS-CoV-2 IGRA became available in the University Hospital laboratory. Therefore, the time between the last booster dose and the study enrollment differed with a median of 21 (13 – 30) weeks. From all PAD patients included, we obtained blood for laboratory tests to evaluate anti-SARS-CoV-2 cellular and humoral responses.

The study was approved by the Bioethics Committee of the Jagiellonian University Medical College (No: 1072.6120.25.2022). All procedures were carried out under the ethical guidelines of the Declaration of Helsinki. Each patient was instructed about the methodology/safety protocol and gave written informed consent to participate in the study.

### 2.2 Levels of anti-SARS-CoV-2 antibodies in serum samples

Serum levels of anti-SARS-CoV-2 IgG were assessed using the anti-SARS-CoV-2 QuantiVac ELISA kit (Euroimmun, Lübeck, Germany) according to the manufacturer’s instructions. This assay allows for the quantitative measurement of IgG against the S1 domain of the SARS-CoV-2 spike protein. The resulting concentration was expressed as binding antibody units per milliliter (BAU/mL) with a detection threshold of 0.8 BAU/mL and positivity threshold ≥35.2 BAU/mL.

### 2.3 Interferon-gamma release assay (IGRA)

The SARS-CoV-2 specific cellular immune response was measured using a Quan-T-Cell SARS CoV-2 assay and ELISA kit (Euroimmun) according to the manufacturer’s instructions. This assay quantifies T-cell mediated response to SARS-CoV-2 spike protein S1. In brief, aliquots of heparinized whole blood were incubated for 24 h (at 37°C) with (1) a pool of peptides derived from S1 protein of SARS-CoV-2, (2) blank (background control), or (3) T-cell mitogen (positive control). The concentration of released IFN-γ was measured in plasma obtained from the blood samples. As per the recommendation of the assay, IGRA was considered positive if the results were above the cut-off point of 200 mIU/mL. The test was interpreted only if the concentration of IFN-γ released upon mitogen stimulation (positive control) was above 400 mIU/mL.

### 2.4 Statistics

Statistical analysis was performed with Statistica 13.3 (TIBCO Software Inc., Palo Alto, CA, USA) and R (version 3.6.1) software. Categorical variables were presented as numbers (percentages), and differences between groups were compared using the Chi^2^ test (Yates corrected). Measurements below the detection thresholds were expressed as the value of the lower cut-off of the assay. As all continuous variables were non-normally distributed (according to the Shapiro-Wilk test), they were presented as medians and 0.25-0.75 quartiles and compared with the Mann-Whitney U test. Correlations were tested with the Spearman rank test. Independent determinants of the increase in SARS-CoV-2 IgG levels and IGRA responses in analyzed subgroups were established in multivariate linear regression models built using a stepwise forward selection procedure, verified by Snedecor’s F-distribution. The R^2^ was checked as a measure of variance. Results with a p-value less than 0.05 were considered statistically significant.

## 3 Results

### 3.1 Characteristics of patients

The study cohort included PAD patients with various types of antibody deficiencies that could be classified into four clinical categories: (1) X-linked agammaglobulinemia (XLA, n = 4), (2) common variable immunodeficiency (CVID, n = 33), (3) isotype defects or IgG subclass deficiency (n = 6), and (4) unclassified IgG deficiency (n = 14), which refers to patients with decreased IgG who did not fulfill the CVID or other PID ESID criteria (21). The detailed clinical and immunological characteristics of each studied subgroup are presented in **Table 1**. The variables shown, such as gender, age of diagnosis, and results of immunologic investigations, correspond to the specific PAD diagnosis. Most patients received IgRT subcutaneously, with satisfactory IgG trough levels obtained in each subset (23).

**Table 1:** Demographic, clinical, and laboratory parameters of the primary antibody deficiency patients.

Parameter	XLA n = 4	CVID n = 33	Isotype defects or IgG subclass deficiency n = 6	Unclassified IgG deficiency n = 14
Age, years	23.5 (20.8 – 28.5)	36.0 (30.0 – 48.5)	57.0 (26.3 – 60.3)	60.0 (47.8 – 66.5)
Sex, male, n (%)	4 (100.0%)	22 (66.7%)	2 (33.3%)	5 (35.7%)
Body mass index, kg/m^2^	25.1 (22.8 – 25.7)	25.2 (20.8 – 28.6)	24.4 (20.2 – 33.5)	24.9 (21.9 – 31.0)
Duration of the disease, years	21.7 (20.2 – 23.0)	7.0 (3.0 –11.0)	10.5 (7.0 – 17.0)	12.0 (9.0 – 16.0)
Age of onset, years	1.8 (1.3 – 4)	30 (15 – 36)	47 (4 – 52)	48.5 (39 – 55)
Mean monthly immunoglobulin dose, g	24.0 (24.0 – 28.5)	30.0 (24.0 – 32.0)	15.8 (16.0 – 22.5)	25.0 (20.0 – 30.5)
Method of immunoglobulin administration, subcutaneous, n (%)	4 (100.0%)	31 (93.9%)	4 (66.7%)	12 (85.7%)
The lowest level of IgG, g/L	1.39 (0.17 – 3.04)	2.58 (1.15 – 3.72)	4.26 (4.16 – 6.8)	5.11 (4.15 – 5.91)
The lowest level of IgA, g/L	0.07 (0.05 – 0.17)	0.07 (0.06 – 0.17)	1.54 (0.51 – 2.07)	1.10 (0.77 – 1.46)
The lowest level of IgM, g/L	0.06 (0.04 – 0.12)	0.17 (0.06 – 0.23)	0.72 (0.53 – 1.18)	0.47 (0.29 – 1.06)
Trough IgG level, g/L	9.05 (8.52 – 9.56)	8.33 (7.03 – 9.30)	9.54 (8.24 – 10.88)	9.17 (7.34 – 9.53)
Peripheral blood B-cell, %	0.0 (0.0 – 0.1)	11.2 (6.0 – 15.2)	10.9 (6.4 – 18.8)	9.9 (3.9 – 12.9)
Peripheral blood CD4+ T-cells, %	43.0 (40.3 – 63.3)	36.7 (29.4 – 41.8)	35.2 (30.3 – 42.4)	38.6 (34.8 – 45.5)
Peripheral blood CD8+ T-cells, %	32.0 (22.8 – 40.2)	34.1 (26.9 – 42.5)	29.3 (24.6 – 31.5)	27.7 (18.4 – 31.0)
Peripheral blood NK cells, %	7.1 (2.5 – 13.8)	9.1 (5.5 – 17.5)	14.7 (13.7 – 26.2)	14.1 (10.0 – 28.3)

Categorical variables are presented as numbers (percentages), and continuous variables as median with a 0.25-0.75 quartile range. CVID, common variable immunodeficiency; n, number; NK, natural killer cells; XLA, X-linked agammaglobulinemia. The lowest IgG, IgA, and IgM levels were detected at primary antibody deficiency diagnosis, that is prior to IgRT.

The majority of patients had internal medicine comorbidities, with the most frequent allergic diseases (e.g., asthma was diagnosed in 26%). Furthermore, over half of the patients (n = 31, 54.4%) suffered from chronic sinusitis. Autoimmune diseases were reported in twelve patients (21.1%). Among them, the most frequent were psoriasis (n = 3) and lupus erythematosus (n = 2). In addition, Sjögren’s syndrome, myasthenia, Hashimoto’s thyroiditis, idiopathic thrombocytopenic purpura, autoimmune hepatitis, alopecia areata, and vitiligo were reported in one single case each. None of the patients received steroids or immunosuppressants at enrollment.

### 3.2 Increased anti-SARS-CoV-2 IgG titers in vaccinated PAD patients

**Figure 1:**
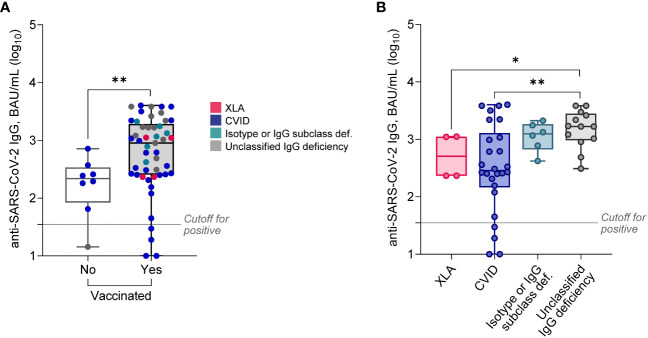
Serum levels of anti-SARS-CoV-2 IgG in PAD patients stratified based on vaccination status **(A)**, or diagnosis (**B**; only vaccinated patients). Data are shown as medians and quartiles. Mann-Whitney test: *p<0.05, **p<0.01. Statistics for XLA and Isotype or IgG subclass deficiency groups are less credible due to the low sample size. The cut-off for positivity in the anti-SARS-CoV-2 IgG assay was ≥35.2 BAU/mL. XLA, X-linked agammaglobulinemia; CVID, common variable immunodeficiency.

First, we compared antiviral humoral responses in patients vaccinated and non-vaccinated against SARS-CoV-2. As it turned out, the majority (91.2%) of all PAD patients had detectable anti-SARS-CoV-2 IgG with an extensive titer range (**Figure 1A**). Positive anti-SARS-CoV-2 IgG results were detected with similar frequency in non-vaccinated (n = 7, 87.5%) and vaccinated patients (n = 45, 91.8%; p = 0.54). Nevertheless, vaccinated patients showed 4.1-fold higher (p = 0.009) median levels of anti-SARS-CoV-2 IgG compared to non-vaccinated (**Table 2**).

**Table 2:** Demographic, clinical, and laboratory parameters in primary antibody deficiency patients stratified according to the vaccination status.

Parameter	Vaccinated n = 49	Non-vaccinated patients n = 8	p-value
Age, years	44.0 (28.0 – 57.0)	36.5 (33.0 – 43.5)	0.12
Sex, male, n (%)	26 (53.1%)	7 (87.5%)	0.15
Body mass index	25.1 (21.9 – 29.1)	22.2 (19.0 – 26.2)	0.12
Mean monthly immunoglobulin dose, g	25 (20 – 30)	30 (29 – 35)	0.08
COVID-19 in medical history, n (%)	15 (30.6%)	3 (37.5%)	0.18
Anti-SARS-CoV-2 IgG, BAU/ml	901.8 (257.1 – 1759.5)	220.2 (122.9 – 314.6)	0.01*
Positive anti-SARS CoV-2 IgG, n (%)	45 (91.8%)	7 (87.5%)	0.54
Positive IGRA, n (%)	44 (89.7%)^&^	5 (62.5%)	0.13
IFN-γ after protein S1 stimulation, mlU/ml	1534 (568 – 3125)^&^	401.5 (46.5 – 740.5)	0.004*
IFN-γ after mitogen, mlU/ml	7725.0 (35330 – 40001.0) ^&^	7423.5 (3287 – 29522)	0.66

Categorical variables are presented as numbers (percentages), and continuous variables as median with a 0.25-0.75 quartile range. Data were compared using the Mann-Whitney test. BAU, binding antibody units; IFN-γ, interferon-gamma; IGRA, interferon-γ release assay; n, number; SARS-CoV-2, severe acute respiratory syndrome coronavirus type 2; an asterisk marks the statistically significant differences; ^&^, reliable data for n = 47 (96%) subjects as IGRA test was not quantifiable in two vaccinated patients with unclassified IgG deficiency. An asterisk marks the statistically significant differences.

The anti-SARS-CoV-2 IgG titers did not depend directly on IgRT mode, i.e., subcutaneous vs. intravenous, or the mean monthly immunoglobulin dose. It also did not rely on the number of vaccine doses or the history of COVID-19 (data not shown). The levels of anti-SARS-CoV-2 IgG correlated positively with the concentration of serum immunoglobulins prior to IgRT (i.e., the lowest concentration), including both IgG (r = 0.34, p = 0.009) and IgA (r = 0.45, p<0.001), but also IgM (r = 0.47, p<0.001) and IgE (r = 0.40, p = 0.005). Surprisingly, anti-SARS-CoV-2 IgG correlated positively with the percentage of peripheral blood NK cells (r = 0.48, p<0.001) but not with the percentage of B-cells (r = -0.17, p = 0.25) or CD4+ and CD8+ T-cells (r = -0.09, p = 0.50 and r = -0.23, p = 0.11, respectively).

CVID patients had approximately 5-fold decreased anti-SARS-CoV-2 IgG levels compared both to unclassified IgG deficiency, and patients with isotype defects or IgG subclass deficiency (**Figure 1B**, **Table 3**). However, these results were unrelated to the main clinical manifestations (data not shown). Interestingly, relatively high anti-SARS-CoV-2 IgG levels were documented in all four XLA males (**Figure 1B**). Additionally, Anti-SARS-CoV-2 IgG levels did not differ between subjects who had COVID-19 in the past, independently of vaccination status (data not shown).

**Table 3:** Demographic, clinical, and laboratory parameters in primary antibody deficiency patients stratified according to the subgroups’ alignment.

Parameter	XLA n = 4	CVID n = 26	Isotype defects or IgG subclass deficiency n = 6	Unclassified IgG deficiency n = 13	CVID vs. unclassified IgG deficiency p-value
Age, years	23.5 (21.5 – 27)	35 (30 – 49)	57 (28 – 60)	64 (50 – 68)	0.001*
Sex, male, n (%)	4 (100.0%)	16 (61.5%)	2 (33.3%)	4 (30.8%)	0.14
Body mass index, kg/m^2^	25.1 (22.9 – 25.7)	25.4 (22.4 – 29.1)	24.4 (20.2 – 33.5)	24.8 (20.7 – 31.0)	0.80
Mean monthly immunoglobulin dose, g	24 (24 – 27)	29 (24 – 32)	16 (16 – 20)	25 (20 – 30)	0.21
Number of vaccinations, n	2 (2 – 3)	3 (2 – 3)	3 (3 – 3)	3 (2 – 3)	0.17
Time from the last vaccination, weeks	19 (5.6 – 35.7)	21.3 (12.7 – 35.7)	15 (10.4 – 18.1)	23.8 (19.1 – 27.4)	0.57
COVID-19 in medical history, n (%)	2 (50.0%)	8 (30.8%)	1 (16.7%)	4 (30.8%)	0.71
Anti-SARS-CoV-2 IgG, BAU/ml	668.2 (232.1-1110.6)	287.2 (156-1077.8)	1249.5 (779.1-1759.5)	1672.8 (1031-2619.5)	0.006*
Positive IGRA test, n (%)	4 (100.0%)	25 (96.1%)	5 (83.3%)	10 (90.9%)^&^	0.88
Concentration of IFN-γ released after spike protein stimulation, mlU/ml	975.6 (829-1410.6)	2334 (803-4737)	442 (330-568)	2413 (966-39991)^&^	0.99
Concentration of IFN-γ after mitogen stimulation, mlU/ml	20950 (5121-37946)	7869 (4189-40001)	4327 (3229-8045)	40001 (2326-40001)^&^	0.54

Categorical variables are presented as numbers (percentages), and continuous variables as median with a 0.25-0.75 quartile range. Data were compared using the U-Mann-Whitney test. BAU, binding antibody units; IFN-γ, interferon-gamma; IGRA, interferon-γ release assay; n, number; SARS-CoV-2, severe acute respiratory syndrome coronavirus type 2; ^&^, reliable results in 11 patients (85%). An asterisk marks the statistically significant differences.

Then, we performed multivariate linear regression models to indicate which parameters independently determined higher anti-SARS-CoV-2 IgG levels either in vaccinated CVID (**Table 4**) or unclassified IgG deficiency patients (**Table 5**). Interestingly, in CVID, lower anti-SARS-CoV-2 IgG levels were observed in those receiving higher monthly immunoglobulin doses and characterized by an increased percentage of B cells and CD8+ T-cells in the lymphocyte subpopulations. In turn, higher levels of the anti-SARS-CoV-2 IgG might be predicted by the increased values of the lowest IgA at PAD diagnosis and the higher percentage of NK and CD4+ T-cells. On the other hand, in those with unspecified antibody deficiency, elevated anti-SARS-CoV-2 IgG levels were independently determined by the higher B-cell percentage and higher concentrations of the lowest IgG, IgM, and IgE, evaluated before IgRT.

**Table 4:** Multiple linear regression model for a relative increase in anti-SARS-CoV-2 IgG levels in vaccinated common variable immunodeficiency (CVID) patients (n = 26).

	β (95% CI)	R^2^
anti-SARS-CoV-2 IgG level, BAU/ml		
Monthly immunoglobulin dose, g	–0.64 (–0.75 to –0.50)	0.79
Peripheral blood B-cell, %	–0.17 (–0.28 to –0.05)	
Peripheral blood CD4+ T-cells, %	0.30 (0.15 to 0.43)	
Peripheral blood CD8+ T-cells, %	–0.13 (–0.24 to –0.01)	
Peripheral blood NK cells, %	0.29 (0.16 to 0.41)	
Number of lymphocytes, number per μl of peripheral blood	0.20 (0.05 to 0.34)	
The lowest level of IgA, g/L	0.49 (0.37 to 0.61)	
Adjustment statistics	F = 9.33, p<0.0001	

BAU, binding antibody units; the lowest IgA was determined at the diagnosis, that is prior to IgRT. Presented variables have been reported as independent determinants, explaining 79% of anti-SARS-CoV-2 IgG levels variability.

**Table 5:** Multiple linear regression model for a relative increase in anti-SARS-CoV-2 IgG levels in vaccinated patients with unclassified IgG deficiency (n=13).

	β (95% CI)	R^2^
anti-SARS-CoV-2 IgG level, BAU/ml		
Peripheral blood B-cell, %	0.47 (0.27 to 0.67)	0.75
The lowest level of IgG, g/L	0.62 (0.42 to 0.81)	
The lowest level of IgM, g/L	0.43 (0.24 to 0.63)	
The lowest level of IgE, g/L	0.52 (0.32 to 0.72)	
Adjustment statistics	F = 6.12, p = 0.01	

BAU, binding antibody units; the lowest IgG, IgM, and IgE levels were determined at the diagnosis, that is prior to IgRT. Presented variables have been reported as independent determinants, explaining 75% of anti-SARS-CoV-2 IgG levels variability.

### 3.3 Vaccinated PAD patients show increased IFN-γ response after *in vitro* stimulation of mononuclear blood cells with SARS-CoV-2 spike antigen

**Figure 2:**
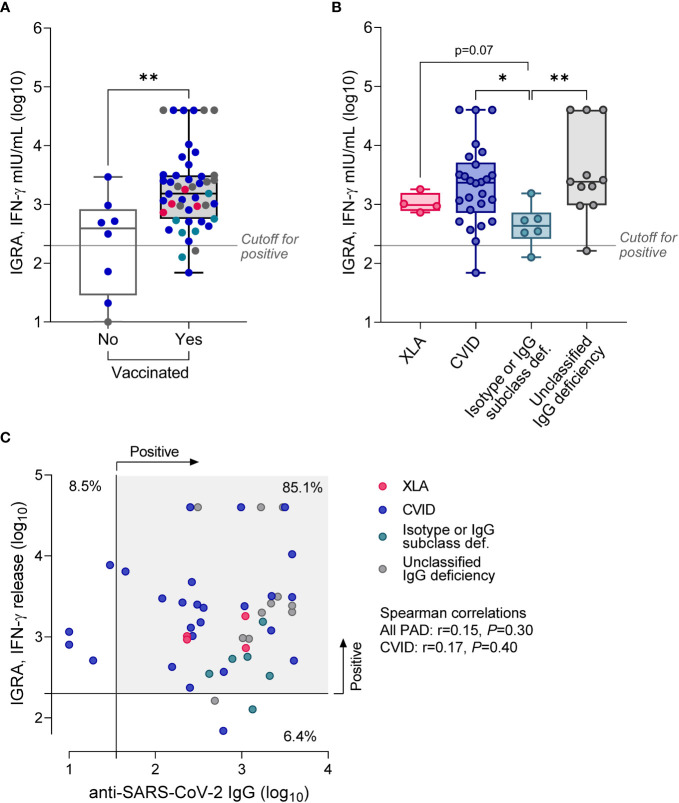
Results of IGRA test in PAD patients stratified according to the vaccination status **(A)**, or diagnosis (**B**; only vaccinated patients). Data are shown as medians and quartiles. Mann-Whitney test: *p<0.05, **p<0.01. Statistics for XLA and Isotype or IgG subclass deficiency groups are less credible due to the low sample size. The cut-off for positivity in the IGRA was >200 mIU/mL. **(C)** No correlation between IGRA results and the level of anti-SARS-CoV-2 IgG. The scatter plot shows results for vaccinated patients (n = 47), in two patients with unclassified IgG deficiency IGRA test was not quantifiable; Clinical groups are assigned with colors. XLA, X-linked agammaglobulinemia; CVID, common variable immunodeficiency.

Next, we analyzed cellular immune response using the IGRA test with SARS-CoV-2 spike protein stimulation. Reliable results, with the correct positive control, were obtained in 55 of 57 patients (96%). Similarly to anti-SARS-CoV-2 IgG antibody levels, positive results were detected with similar frequency in non-vaccinated (n = 5, 63%) vs. vaccinated patients (n = 44, 89.7%; p = 0.13, **Table 2**). However, vaccinated patients showed 3.8-fold higher IFN-γ production in the IGRA than non-vaccinated (**Figure 2A**). There were five patients who were not vaccinated and did not suffer from SARS-CoV-2 infection in the past. Interestingly, the IGRA test was positive in three of them, suggesting asymptomatic viral exposure (data not shown).

Further subgroup analysis revealed considerable heterogeneity of IGRA results in CVID and unclassified IgG deficiency, yet with almost the same median concentration of released IFN-γ in the two subgroups (**Figure 2B**; **Table 3**). Patients with CVID showed 2.4-fold higher (p = 0.23) production of IFN-γ compared to XLA and 5.4-fold higher (p = 0.016) than patients with isotype defects and IgG subclass deficiency. Similar data were recorded for patients with unclassified IgG deficiency; that is, median IGRA results in this group were 2.5-fold higher (p = 0.078) compared to XLA and 5.5-fold higher (p = 0.009) compared to patients with isotype defects and IgG subclass deficiency. It is necessary to mention that data regarding the XLA group are less credible due to the meager sample size. IFN-γ production in response to SARS-CoV-2 spike antigen did not correlate with the anti-SARS-CoV-2 IgG levels (r = 0.14, p = 0.38), though over 80% of vaccinated patients had both tests positive (**Figure 2C**). Similarly, IGRA results were not directly related to the number of vaccination doses and mean monthly immunoglobulin dose, or immunological laboratory parameters in direct evaluation (data not shown).

Regarding multiple regression analyses, we could build models only for vaccinated CVID patients. The strongest one is presented in **Table 6**. Interestingly, as shown, all included factors predicted IGRA responses negatively. Among them, the most important was the impact of time since the last vaccination and, surprisingly, the lowest IgG levels prior to IgRT. Furthermore, similar to anti-SARS-CoV-2 IgG levels, higher IGRA responses were documented in those requiring lower IgRT monthly doses.

**Table 6:** Multiple linear regression model for a relative increase in IFN-γ response assay (IGRA) in vaccinated common variable immunodeficiency (CVID) patients (n = 26).

	β (95% CI)	R^2^
Concentration of IFN-γ released after spike protein stimulation, mlU/ml		
Monthly immunoglobulin dose, g	–0.22 (–0.38 to –0.07)	0.50
Time from the last vaccination, weeks	–0.49 (–0.65 to –0.33)	
Peripheral blood CD8+ T-cells, %	–0.28 (–0.44 to –0.12)	
The lowest level of IgG, g/L	–0.47 (–0.62 to –0.32)	
Adjustment statistics	F = 5.43, p=0.003	

IFN-γ, interferon-γ; IGRA, interferon-γ release assay; the lowest IgG was determined at the diagnosis prior to IgRT. Presented variables have been reported as independent determinants, explaining 50% of IGRA variability.

## 4 Discussion

In the present study, using IGRA assay with SARS-CoV-2 spike 1 antigen stimulation, we demonstrate an effective cellular immune response in PAD patients vaccinated against SARS-CoV-2. The data regarding cellular immunity to that virus in PID are still limited. Furthermore, they usually include heterogeneous cohorts and experimental research assays (16, 24–26). For example, Oyaert et al. (24) analyzed humoral and cellular immune responses three months after the second vaccine dose in different immunocompromised patients, including 57 patients with PID. They documented positive humoral responses in 89.1% of PID patients but cellular responses in only 47.3-63.6% of patients, depending on the antigen used for *in vitro* cell stimulation in that experimental assay. Interestingly, in a subgroup of patients on IgRT, they demonstrated a reduced seroconversion rate and a significant positive correlation between the B-cell count and the titer of anti-SARS-CoV-2 IgG, similar to our data from subgroup analysis. In turn, Hagin et al. (25) evaluated cellular immune response in 26 PID patients, including 4 with XLA and 12 with CVID, using an experimental ELISpot assay. This test estimated IL-2 and IFN-γ secretion in response to pool of peptides derived from SARS-CoV-2 S or M proteins. The authors demonstrated positive cellular response to S-peptides in 73.1% of patients, including all four with XLA. Similar resuts were reported in small cohorts of PID patients (mostly with PAD) in the study by Pham et al. (16), who showed 77.4% of positive responses using IGRA test with S, N, and M peptides and in the study by Murray et al. (26) who documented 89% of positive cellular responses. In our data, over 90% of PAD patients showed positive IGRA test results, which confirms significant T-cell viral responses even in PAD patients with significant antibody production defects. Additionally, vaccinated PAD patients showed 3.8-fold higher IFN-γ release after *in vitro* stimulation with viral antigen and 4.1- fold higher levels of anti-SARS-CoV-2 IgG antibodies than non-vaccinated. That may suggest that vaccination resulted in the production of their own virus-specific antibodies, added to the pool of protective antibodies already supplemented with immunoglobulin preparations during IgRT. The positive correlation between the titers of anti-SARS-CoV-2 IgG and the lowest immunoglobulin levels prior IgRT supports indirectly this observation.

Interestingly, even patients with X-linked agammaglobulinemia had relatively high levels of anti-SARS-CoV-2 IgG. That is an interesting finding, as XLA diagnosis was confirmed by detecting functional mutations in the *BTK* gene, so the production of specific immunoglobulins should be severely impaired. Indeed, in a recent study by Hagin et al. (25), XLA patients failed to produce anti-S protein antibodies two weeks after a booster dose of mRNA vaccine. One explanation for that discrepancy could be a genetic variant resulting in a trace production of immunoglobulins (27), or all measured immunoglobulins came indeed from IgRT (28). However, even if we assume that all virus-specific immunoglobulins in XLA patients were supplemented, positive IGRA results indicate a robust cellular response, an additional benefit from COVID-19 vaccination.

Another issue that needs to be discussed is a lower level of anti-SARS-CoV-2 IgG in some CVID patients who showed adequate IGRA response. CVID is a complex disease that refers to the variable combined humoral and cellular immunodeficiency (29, 30). Interestingly, in multivariable logistic regression analysis of that group, anti-SARS-CoV-2 IgG levels, but also IGRA responses, were negatively determined by mean monthly immunoglobulin doses. That observation suggests that more severe CVID cases requiring higher regular doses of IgRT, produce less of their own antiviral antibodies and are characterized by diminished cellular responses. Contrary to the higher immunoglobulin levels at PAD diagnosis, which determine higher anti-SARS-CoV-2 IgG levels, but at the same time, lower IFN-γ release in cellular immunity assay, as shown in multivariable analysis. Furthermore, interestingly, the multivariable regression models of anti-SARS-CoV-2 IgG levels suggest that different immunologic mechanisms prevail in specific IgG production in CVID and unclassified IgG deficiency patients. In CVID, CD4+ T and NK cells might be important, while in unclassified IgG deficiency, B-cells.

Thus, our results suggest that impaired cellular immunity and a more severe antibody deficiency in some CVID patients might result in lower anti-SARS-CoV-2 IgG levels after vaccination. Therefore, these patients could benefit from vaccination mainly through enhanced cellular immune responses. The cellular immune response mediated by cytotoxic T-cells is crucial for eliminating infected cells and limiting viral replication (31). However, immunocompromised patients often exhibit poor that response (32). For example, Stanevich et al. (33) demonstrated that impairment in the CD8+ T-cell compartment plays a vital role in acquiring new mutations by the virus that reduce the binding to HLA class I, resulting in further escape from CD8+ T-cell control. Furthermore, T-cells, particularly CD4+, stimulate humoral responses via cytokine production, which might also be impaired in PID (34). Altogether, cellular and humoral mechanisms interact to combat viral infections, and overall these data suggest that a subset of CVID patients with impaired humoral and cellular responses may require a more intensive vaccination schedule than other PAD, particularly that time from the last booster dose determined IGRA results negatively in multiple regression model. However, more extensive observational and experimental studies are needed to verify that hypothesis.

We also noticed decreased IFN-γ production in the IGRA test in patients with isotype defects or IgG subclasses deficiency, suggesting that impairment in cellular immunity may essentially contribute to the immune deficiency in this clinical subtype. That may also explain our observation that these subjects, characterized by relatively minor and potentially underestimated antibody deficiency, have similar clinical manifestations to other PAD subtypes, including CVID, and usually require long-term IgRT. Nevertheless, these conclusions need to be validated in a larger study cohort due to the meager sample size.

### 4.1 Study limitation

The major limitation of our study is the relatively small sample size and the lack of long-term follow-up. However, we enrolled only PAD patients with confirmed diagnoses who remained on long-term and regular IgRT for at least 12 months We did not measure anti-SARS-CoV-2 IgG titers in immunoglobulin products; thus, we cannot assess whether it correlated with those evaluated in PAD patients. Furthermore, we analyzed the IGRA test only once, and the patients differed to some extent with the time from the last booster dose. Yet vaccination-related variables did not impact IGRA measurements; thus, we believe they should not significantly influence results.

## 5 Conclusions

In conclusion, the present study demonstrates that PAD patients show satisfactory *in vitro* cellular immune responses after SARS-CoV-2 vaccination, including those with more severe antibody deficiency, such as XLA and CVID. Nevertheless, more observational and experimental studies with longer follow-ups are needed to determine whether SARS-CoV-2 vaccination is crucial to assess the persistence of cellular immunity and protect against COVID-19. Furthermore, detecting anti-SARS-CoV-2 IgG antibodies alone in PAD patients is not eligible, as these antibodies are present in the IgRT. Therefore, assessing cellular immune responses, such as the IFN-γ release assay, becomes a mainstay in determining the functional immune response in patients with immune deficiencies.

## Data availability statement

The original contributions presented in the study are included in the article. Further inquiries can be directed to the corresponding author.

## Ethics statement

The studies involving humans were approved by Bioethics Committee of the Jagiellonian University Medical College. The studies were conducted in accordance with the local legislation and institutional requirements. The participants provided their written informed consent to participate in this study.

## Author contributions

DM: Conceptualization, Investigation, Methodology, Project administration, Resources, Software, Writing – original draft. RD: Conceptualization, Data curation, Formal Analysis, Methodology, Software, Visualization, Writing – original draft. AD: Data curation, Methodology, Resources, Software, Writing – review & editing. AG: Data curation, Methodology, Resources, Software, Writing – review & editing. BJ: Conceptualization, Data curation, Formal Analysis, Software, Validation, Visualization, Writing – review & editing. MC: Conceptualization, Investigation, Resources, Writing – review & editing. AP: Conceptualization, Investigation, Resources, Writing – review & editing. AM: Conceptualization, Investigation, Resources, Writing – review & editing. LZ: Formal Analysis, Validation, Visualization, Writing – review & editing. SB: Conceptualization, Formal Analysis, Funding acquisition, Project administration, Resources, Supervision, Validation, Visualization, Writing – review & editing.
